# Morphometric Analysis of the Maxillary Sinus Using CT-Based Air-Cavity Reconstruction: A Retrospective Study

**DOI:** 10.3390/jcm15166173

**Published:** 2026-08-09

**Authors:** Pelin İsmailoğlu, Alp Bayramoğlu, Tuğba Yemiş, Muhammed Sadıkzade, Nevnihal Akbaytürk, Özlem Çelebi Erdivanlı

**Affiliations:** 1 Department of Anatomy, School of Medicine, Recep Tayyip Erdoğan University, Rize 53020, Türkiye; pelin.ismailoglu@erdogan.edu.tr; 2 Department of Anatomy, School of Medicine, Acibadem Mehmet Ali Aydinlar University, Istanbul 34752, Türkiye; 3 Department of Otorhinolaryngology, School of Medicine, Recep Tayyip Erdoğan University, Rize 53020, Türkiye; tugba.yemis@erdogan.edu.tr (T.Y.); mhsd2021@gmail.com (M.S.); drozlemcelebi@hotmail.com (Ö.Ç.E.); 4 Department of Anatomy, School of Medicine, Giresun University, Giresun 28100, Türkiye; nevnihal.akbayturk@giresun.edu.tr

**Keywords:** maxillary sinus, morphometry, anatomical variation, computed tomography, septa, air-cavity reconstruction

## Abstract

**Background/Objectives**: This study aimed to characterize the three-dimensional morphology of the aerated maxillary sinus using CT-based air-cavity reconstruction and to evaluate its relationship with selected morphometric parameters, septa characteristics, bilateral symmetry, and dental root extension. **Methods**: This retrospective study included 125 paranasal computed tomography (CT) examinations from 61 males, 64 females (mean age 37.04 ± 15.37 years), corresponding to 250 maxillary sinuses. Three-dimensional air-cavity segmentation was performed using Mimics software following a standardized Hounsfield unit-based thresholding protocol. Morphological patterns of the aerated cavity were classified according to internal compartmentalization and associated morphological features. Morphometric measurements, including ostium-to-sinus-floor distance, axial sinus dimensions, multiplanar septal characteristics, and dental root extension, were recorded and statistically analyzed. **Results**: Unilocular morphology represented the predominant configuration on both sides (right: 55.2%; left: 53.6%), whereas bilocular and multilocular patterns were less common. Morphologically more complex configurations demonstrated a higher frequency of coronal and axial septa than simple unilocular cavities. Side-to-side differences were identified for ostium-to-sinus-floor distance (*p* = 0.050), axial longitudinal sinus length (*p* = 0.027), and pole measurements (*p* < 0.001). Dental root extension was more frequent on the left side (16.0%) than on the right (6.4%; *p* = 0.002). Male participants exhibited greater ostium-to-sinus-floor distances and larger axial sinus dimensions than females (all *p* < 0.05), whereas age showed only a weak negative correlation with left axial transverse sinus length (ρ = −0.191, *p* = 0.032). Inter-observer agreement for the proposed 18-category three-dimensional morphological classification was substantial (Cohen’s κ = 0.726 for the right side and κ = 0.717 for the left side; both *p* < 0.001). **Conclusions**: CT-based three-dimensional air-cavity reconstruction enabled detailed evaluation of the internal morphology of the aerated maxillary sinus. The observed variability in morphological patterns, septa distribution, and selected morphometric characteristics suggests that assessment of the aerated cavity may complement conventional morphometric evaluation of the maxillary sinus. These findings provide a descriptive anatomical reference that may support future radiological, anatomical, and computational investigations of maxillary sinus morphology.

## 1. Introduction

The maxillary sinus (MS) is the largest of the paranasal sinuses and exhibits considerable anatomical variability in its size, shape, degree of pneumatization, and internal bony architecture [1,2]. Because of its close anatomical relationship with the orbit, nasal cavity, alveolar process, and maxillary dentition, detailed knowledge of maxillary sinus anatomy is essential for endoscopic sinus surgery, dental implant procedures, sinus floor elevation, and other maxillofacial interventions [3,4]. Consequently, numerous radiological studies have investigated maxillary sinus dimensions, septa, and anatomical variations to improve preoperative assessment and reduce surgical complications [5].

Among these anatomical variations, maxillary sinus septa have received particular attention because of their potential influence on surgical access and sinus floor augmentation procedures [6]. Previous studies have primarily focused on the prevalence, location, orientation, and developmental origin of septa [7,8]. Several hypotheses have been proposed regarding their formation, including persistence of developmental bony partitions during pneumatization, craniofacial growth patterns, tooth development, and masticatory loading [6,9,10]. Despite these investigations, most studies have evaluated septa as isolated anatomical structures rather than as components influencing the overall three-dimensional architecture of the sinus cavity [6,9,10]. From an anatomical perspective, however, septa are not merely discrete bony projections; they may alter the configuration of the aerated cavity by creating grooves, lobulations, partial partitions, or multiple communicating compartments [5,11]. Consequently, the presence, orientation, and extent of septa may contribute to three-dimensional remodeling and compartmentalization of the maxillary sinus air space [6,12]. While the prevalence of septa has been extensively documented, considerably less attention has been paid to how these structures reshape the internal morphology of the aerated sinus cavity as a whole [6,12].

While multidetector computed tomography (CT) remains the reference imaging modality for evaluating the paranasal sinuses, conventional two-dimensional assessment is inherently limited in depicting the complex internal geometry of the maxillary sinus [13]. Segmentation-based reconstruction has therefore emerged as an important tool for extracting quantitative and morphological information from CT datasets, allowing improved visualization of sinus anatomy for diagnostic evaluation, surgical simulation, anatomical education, and morphometric analysis [14]. Manual, semi-automatic, and automatic segmentation approaches have all been described, reflecting the increasing importance of three-dimensional reconstruction in sinonasal imaging [7]. In contrast, the present study uses CT-based segmentation as a methodological tool for anatomical characterization of the aerated maxillary sinus. For example, Morgan et al. developed a convolutional neural network capable of automatically segmenting the maxillary sinus on CBCT images with high accuracy, substantially reducing segmentation time while maintaining anatomical fidelity [2]. These advances demonstrate that reliable three-dimensional reconstruction has become increasingly feasible for clinical and research applications [6,7,15,16].

Although three-dimensional segmentation of the paranasal sinuses has become increasingly feasible with commercial software and artificial-intelligence-based approaches, previous studies have primarily emphasized segmentation performance, volumetric assessment, or algorithm validation rather than comprehensive anatomical characterization of the aerated maxillary sinus [2,13,15]. For example, Cellina et al. demonstrated that different segmentation platforms can generate three-dimensional reconstructions of the paranasal sinuses and provide volumetric information, highlighting the potential clinical utility of segmentation for diagnostic assessment [13]. Likewise, Morgan et al. developed and validated a convolutional neural network for automatic CBCT-based maxillary sinus segmentation, focusing on segmentation accuracy, efficiency, and virtual model generation rather than anatomical morphology [2]. Consequently, despite advances in segmentation methodology, there remains no standardized descriptive framework for systematically characterizing the three-dimensional internal morphology of the aerated maxillary sinus.

Current anatomical investigations of the maxillary sinus predominantly emphasize linear dimensions, sinus volume, odontogenic relationships, or septa arising from the sinus floor because of their importance in implant dentistry and sinus augmentation procedures [5,17]. While these structures are clinically important, they represent only one component of the internal architecture of the sinus [9]. Three-dimensional air-cavity reconstruction may additionally reveal superiorly oriented bony projections extending inferiorly from the orbital floor, contributing to internal compartmentalization of the aerated cavity [5,17,18]. Further characterization of the anatomical features of the maxillary sinus, including superior septa, may contribute to a more comprehensive understanding of maxillary sinus morphology [5,17,18]. Likewise, the internal configuration of the aerated cavity itself has received relatively limited attention [19]. Three-dimensional reconstruction may reveal lobulations, deep grooves, compartment-like extensions, surface irregularities, and varying degrees of bilateral asymmetry, suggesting that the maxillary sinus is not invariably a uniformly smooth pyramidal cavity [11]. Beyond its structural anatomy, the maxillary sinus functions as an aerated cavity in which ventilation and mucociliary clearance contribute to physiological homeostasis [11,20]. Recent advancements in computational fluid dynamics have demonstrated that these internal airflow dynamics are highly sensitive to individual anatomical configurations [19]. Previous computational fluid dynamics studies have suggested that common anatomical variations within the sinonasal complex may alter local airflow characteristics [11]. Because airflow characteristics are influenced by the geometry of the aerated cavity, detailed three-dimensional morphological characterization may provide an anatomical basis for future computational fluid dynamics and functional investigations [3,19,21].

Accordingly, the present study was designed as a descriptive anatomical investigation combining CT-derived morphometric analysis with three-dimensional air-cavity reconstruction. Using standardized CT-based segmentation as an anatomical reconstruction tool, the internal morphology of the aerated maxillary sinus was systematically characterized through a reproducible three-dimensional morphological classification, while assessing morphological patterns, bilateral symmetry, ostium-to-sinus-floor distance, axial dimensions, multiplanar septal characteristics, and dental root extension. By integrating conventional morphometric measurements with three-dimensional morphological assessment, this study aims to provide a descriptive anatomical framework that may complement conventional radiological evaluation, facilitate preoperative anatomical assessment, and serve as a basis for future investigations of maxillary sinus morphology and its potential functional implications.

## 2. Materials and Methods

### 2.1. Study Design

The study was approved by the Recep Tayyip Erdoğan University Non-Interventional Clinical Research Ethics Committee (Approval No. 2026/176). The study was conducted in accordance with the ethical principles of the Declaration of Helsinki.

Paranasal CT examinations were retrospectively retrieved from the digital radiology archive of Rize Training and Research Hospital. The examinations had originally been obtained for routine clinical indications, including headache, dizziness, facial pain, and pre-procedural evaluation, and were not acquired specifically for research purposes. An initial archive screening identified approximately 300 paranasal CT examinations. All CT examinations were independently evaluated by an anatomist with nine years of experience in anatomy and an otorhinolaryngologist with seven years of clinical experience. The two experts performed all morphometric measurements and selected the CT datasets from the institutional archive according to predefined anatomical suitability criteria for three-dimensional morphological analysis. Eligible CT datasets were required to demonstrate complete bilateral visualization of the maxillary sinuses with adequate isotropic image quality (slice thickness: 0.625 mm), enabling reliable three-dimensional air-cavity segmentation and morphometric assessment.

Anatomically intact maxillary sinuses were selected as those without previous sinonasal surgery, maxillofacial trauma or fracture, congenital craniofacial anomalies, neoplastic lesions, or other structural abnormalities affecting the maxillary sinus. CT examinations without radiological evidence of inflammatory disease were selected as those showing no complete or partial maxillary sinus opacification, air-fluid level, or diffuse mucosal thickening that could alter the aerated sinus cavity or interfere with three-dimensional segmentation. Minimal incidental mucosal thickening that did not distort the osseous boundaries of the maxillary sinus or compromise three-dimensional air-cavity segmentation was not considered an exclusion criterion. Examinations with incomplete anatomical coverage or image artifacts that could compromise three-dimensional reconstruction or morphometric measurements were excluded. Following the application of the eligibility criteria, 125 paranasal CT examinations (61 males and 64 females) were included in the study. Consequently, 250 maxillary sinuses (right and left) were analyzed using conventional morphometric measurements in combination with CT-based three-dimensional air-cavity segmentation and morphological analysis.

**Figure 1:**
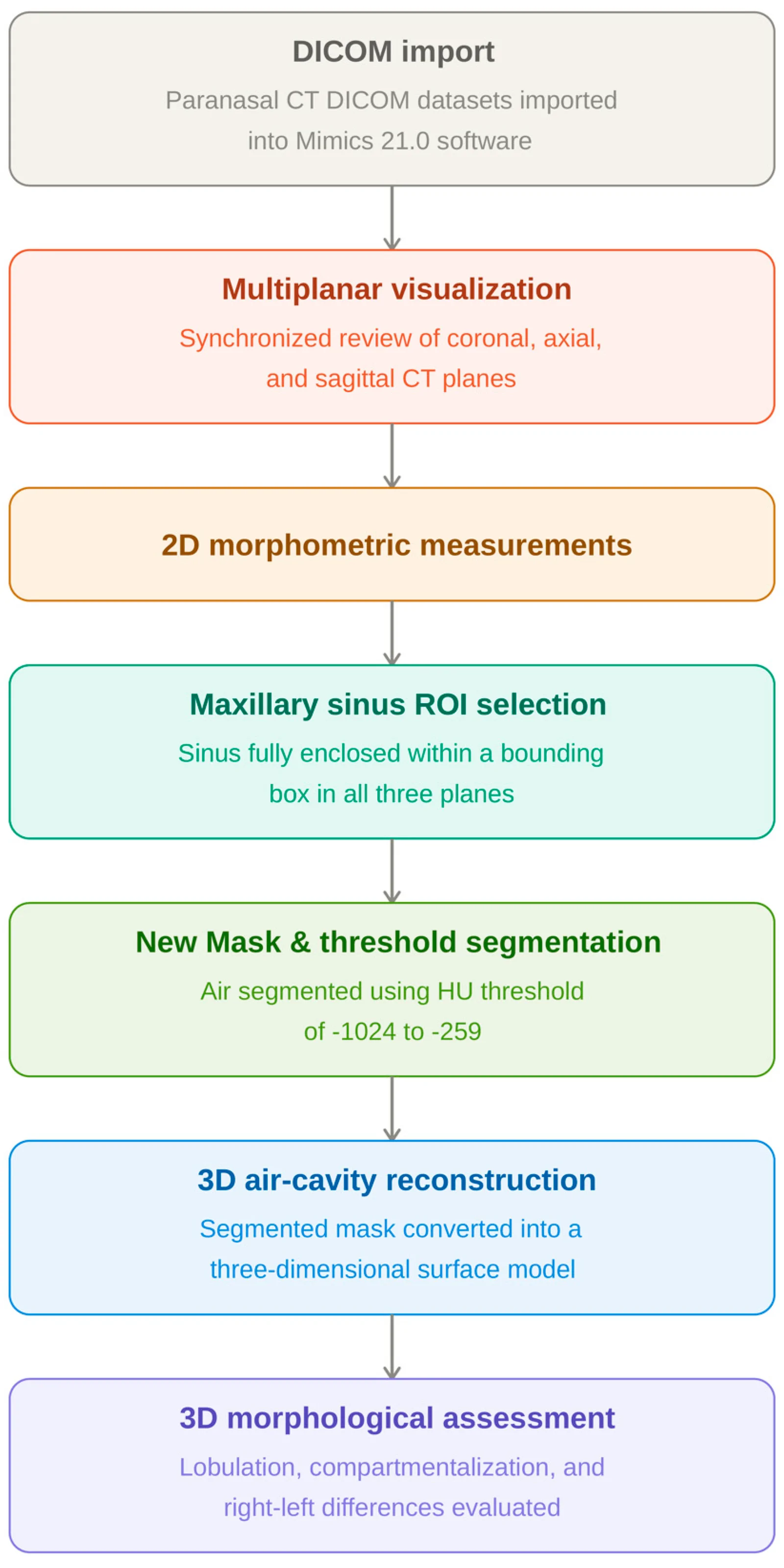
Overall workflow of CT-based morphometric analysis and three-dimensional air-cavity reconstruction of the maxillary sinus. Paranasal CT DICOM datasets were imported into Mimics 21.0 software (Materialise NV, Leuven, Belgium), reviewed using synchronized multiplanar visualization, subjected to conventional two-dimensional morphometric measurements, followed by region-of-interest selection, threshold-based air segmentation, three-dimensional reconstruction, and qualitative morphological assessment.

The Digital Imaging and Communications in Medicine (DICOM) datasets were imported into Mimics 21.0 software (Materialise NV, Leuven, Belgium). Following image loading, multiplanar reconstruction was simultaneously displayed in the axial, coronal, and sagittal planes, allowing synchronized navigation through all three orthogonal views. The overall image-processing and three-dimensional reconstruction workflow leading to this classification is illustrated in Figure 1.

### 2.2. CT-Based Air-Cavity Segmentation and Three-Dimensional Morphological Assessment

Three-dimensional segmentation of the maxillary sinus air cavity was performed using Mimics software (Materialise NV, Leuven, Belgium). The DICOM datasets were imported into the software, and an initial mask was manually created by selecting only the air-containing regions corresponding to the bilateral maxillary sinuses together with the adjacent nasal cavity. This preliminary mask served as the basis for subsequent three-dimensional reconstruction.

**Figure 2:**
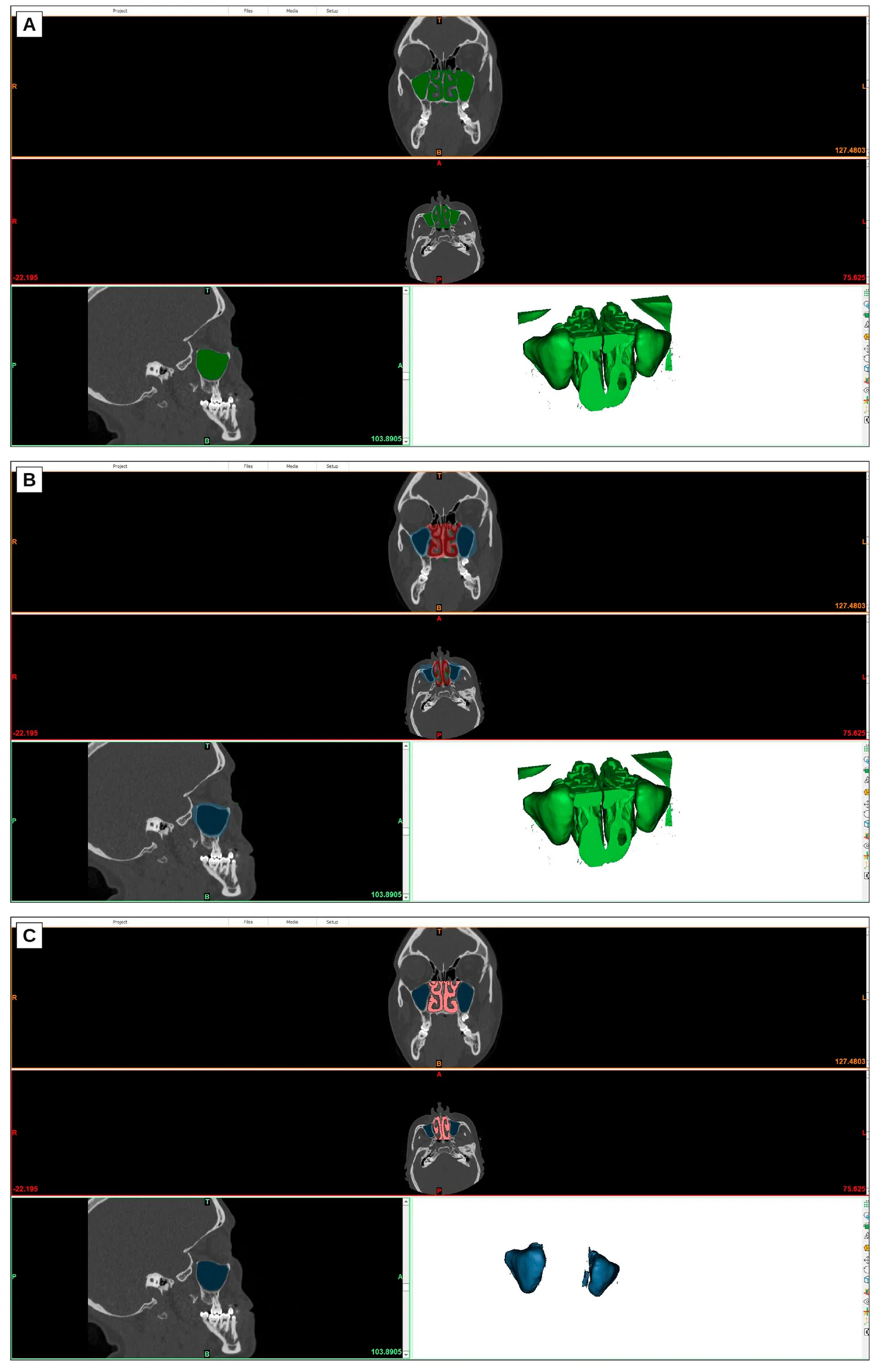
CT-based workflow for three-dimensional reconstruction of the aerated maxillary sinus. Representative images demonstrate sequential processing in Mimics software: (**A**) import of the paranasal CT dataset, region-based segmentation of the maxillary sinus; (**B**) segmentation of adjacent nasal cavity and threshold-based isolation of the aerated cavity (−1024 to −259 HU); and (**C**) generation of the final three-dimensional model. Abbreviations: R, right; L, left; T, top; B, base.

To isolate the aerated cavity, a predefined Hounsfield Unit (HU) range of −1024 to −259 HU, corresponding to air-containing structures, was applied uniformly to all CT datasets. This threshold range was selected based on previously published studies using Mimics software for paranasal sinus air-cavity segmentation, which have reported upper threshold values ranging from approximately −260 HU to −200 HU for three-dimensional reconstruction of sinonasal air spaces [2,22,23]. No modification of the threshold values was required for individual examinations. The generated masks were visually inspected in the axial, coronal, and sagittal planes to confirm complete inclusion of the aerated maxillary sinus cavity. The segmentation masks were then directly converted into three-dimensional surface models. No region-growing procedure or manual mask editing was performed (Figure 2).

**Figure 3:**
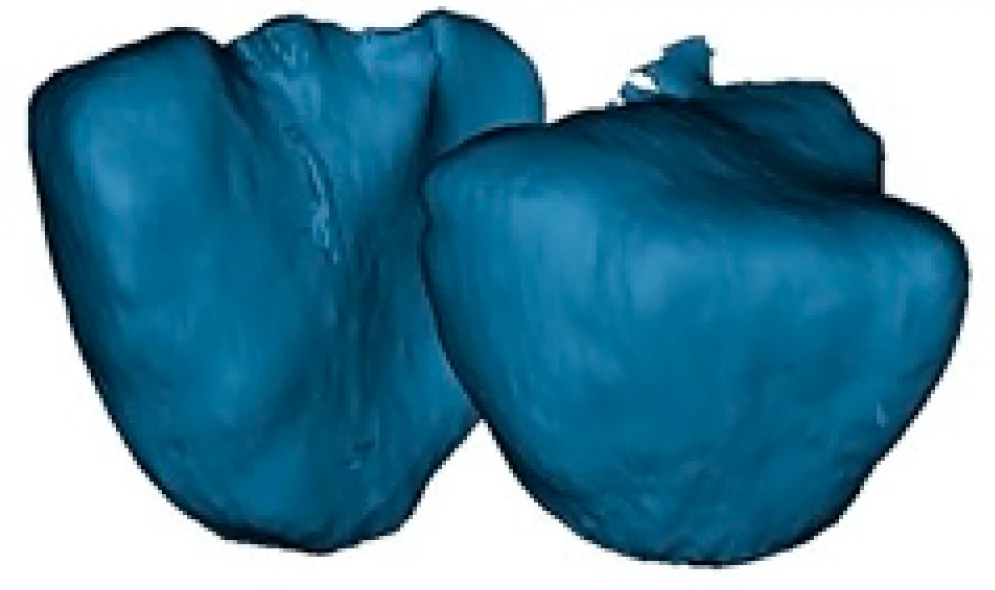
Representative three-dimensional reconstruction of the aerated maxillary sinuses following CT-based segmentation. The reconstructed air cavities were used for subsequent qualitative morphological classification and morphometric analyses.

The finalized segmentation masks were then converted into three-dimensional surface models representing the aerated maxillary sinus cavity (Figure 3). These patient-specific three-dimensional reconstructions were subsequently used for morphological classification and qualitative assessment of the internal air-cavity configuration.

### 2.3. Morphometric Measurements

**Figure 4:**
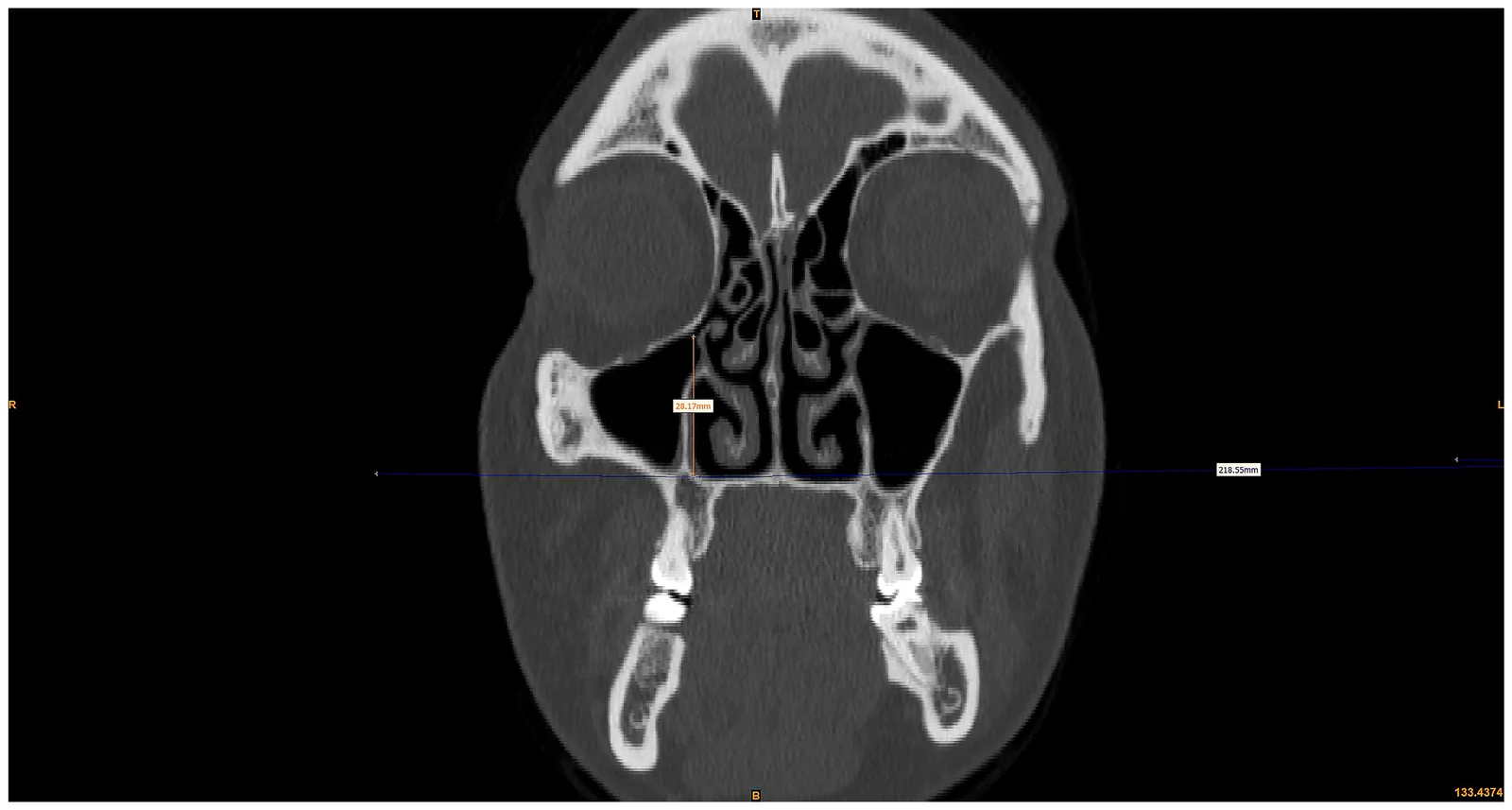
Measurement of the ostium-to-sinus-floor distance (OSFD) on the coronal CT image. The linear distance was measured from the inferior margin of the natural maxillary ostium to the lowest point of the maxillary sinus floor on the coronal section demonstrating the natural ostium most clearly. Abbreviations: R, right; L, left; T, top; B, base.

Morphometric measurements were performed using synchronized multiplanar CT images displayed simultaneously in the coronal, axial, and sagittal planes. Predefined anatomical landmarks and imaging criteria were applied consistently across all examinations. For each maxillary sinus, the coronal section demonstrating the natural maxillary ostium most clearly was identified. The ostium-to-sinus-floor distance (OSFD) was measured as the linear distance between the inferior margin of the natural ostium and the lowest point of the maxillary sinus floor using the distance measurement tool (Figure 4). The natural maxillary ostium was recorded as present when it was clearly identified on the selected coronal CT section, covered when it was obscured by adjacent soft tissue or mucosal coverage, and not visualized when it could not be identified on the available CT images despite careful multiplanar evaluation. The term not visualized refers to radiological non-visualization rather than true anatomical absence of the natural ostium.

The same standardized coronal section was subsequently used to evaluate the presence of coronal septa, and when present, their anatomical location and maximum vertical length were recorded. Measurements were obtained independently for the right and left maxillary sinuses.

**Figure 5:**
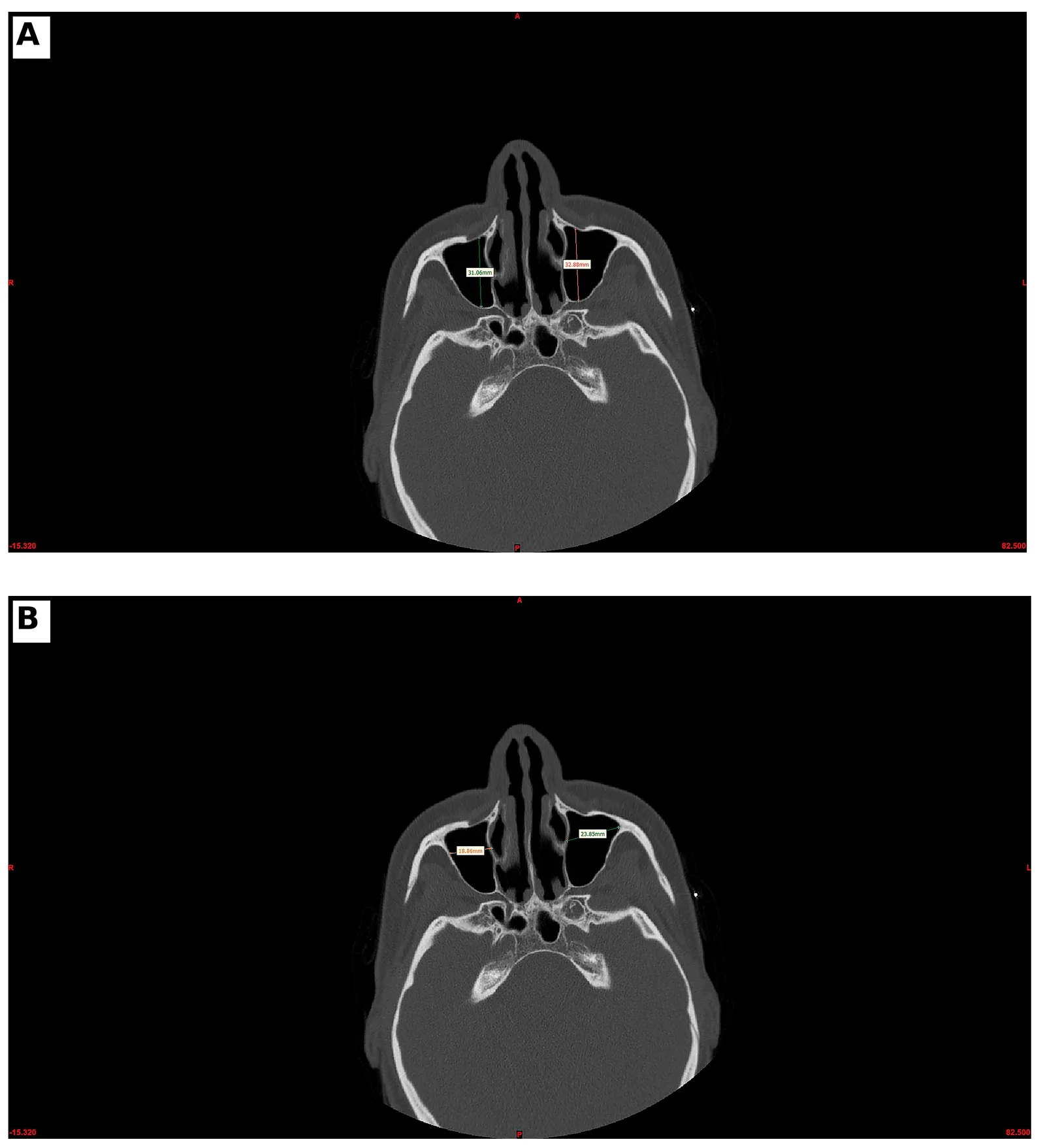
Axial morphometric measurements of the maxillary sinus. (**A**) Maximum anteroposterior diameter measured on the axial CT section. (**B**) Maximum transverse diameter measured on the axial section showing the largest cross-sectional area of the maxillary sinus. Abbreviations: A, anterior; P, posterior; R, right; L, left.

Subsequently, the axial CT section demonstrating the largest cross-sectional area of each maxillary sinus was identified. On this standardized image, the maximum longitudinal (anteroposterior) diameter was measured as the greatest linear distance between the anterior and posterior inner boundaries of the aerated maxillary sinus cavity. The maximum transverse (mediolateral) diameter was measured as the greatest linear distance between the medial and lateral inner boundaries of the aerated maxillary sinus cavity on the same axial section using the linear distance tool. Measurements were obtained independently for the right and left maxillary sinuses using the same anatomical landmarks and measurement criteria (Figure 5A,B).

**Figure 6:**
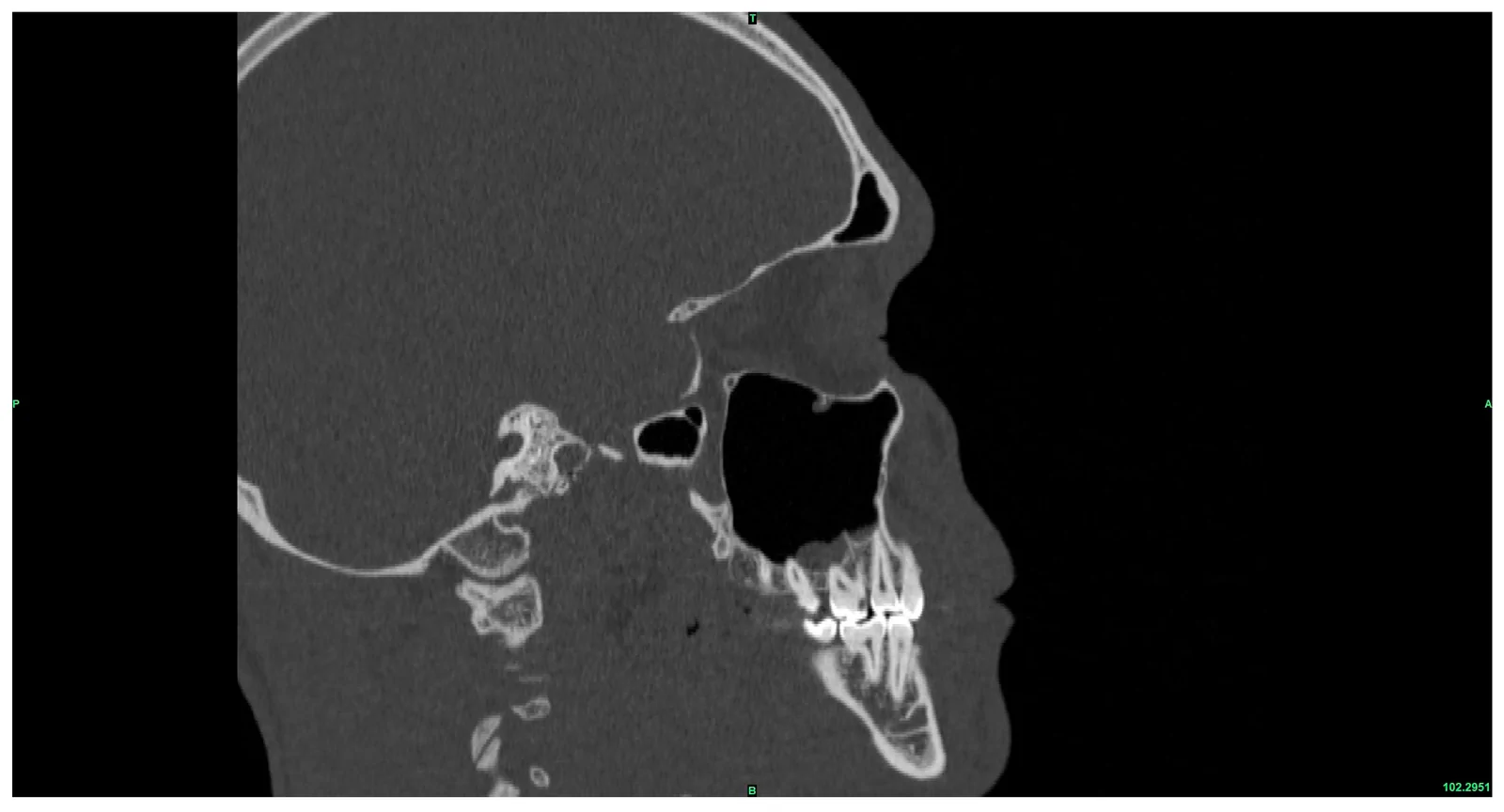
Sagittal evaluation of maxillary sinus anatomy. Representative sagittal CT image illustrating assessment of septal morphology and the relationship between posterior maxillary tooth roots and the maxillary sinus floor. Abbreviations: A, anterior; P, posterior; T, top; B, base.

Following completion of the morphometric measurements, the entire multiplanar CT dataset was systematically reviewed to further characterize septal morphology and anatomical variation. Coronal, axial, and sagittal images were evaluated together to determine the anatomical origin, spatial orientation, and distribution of septa (Figure 6). Septa were classified according to their site of origin from the orbital floor (superior wall), medial wall, lateral wall, anterior wall, posterior wall, maxillary sinus floor, or tooth root, and their relationship to the internal configuration of the maxillary sinus was documented. Sagittal CT images were systematically evaluated using the same standardized anatomical criteria to assess the presence, distribution, and extent of sagittal septa. Dental root extension was evaluated by recording protrusion of one or more posterior maxillary tooth roots beyond the sinus floor into the maxillary sinus cavity. Finally, the three-dimensional morphology of the aerated maxillary sinus was classified according to the degree of internal compartmentalization and associated morphological features, including lobulations, grooves, and isolated compartments. The proposed CT-based morphological classification is presented in Table 1. All observations were recorded independently for the right and left maxillary sinuses.

### 2.4. Pole Measurements

**Figure 7:**
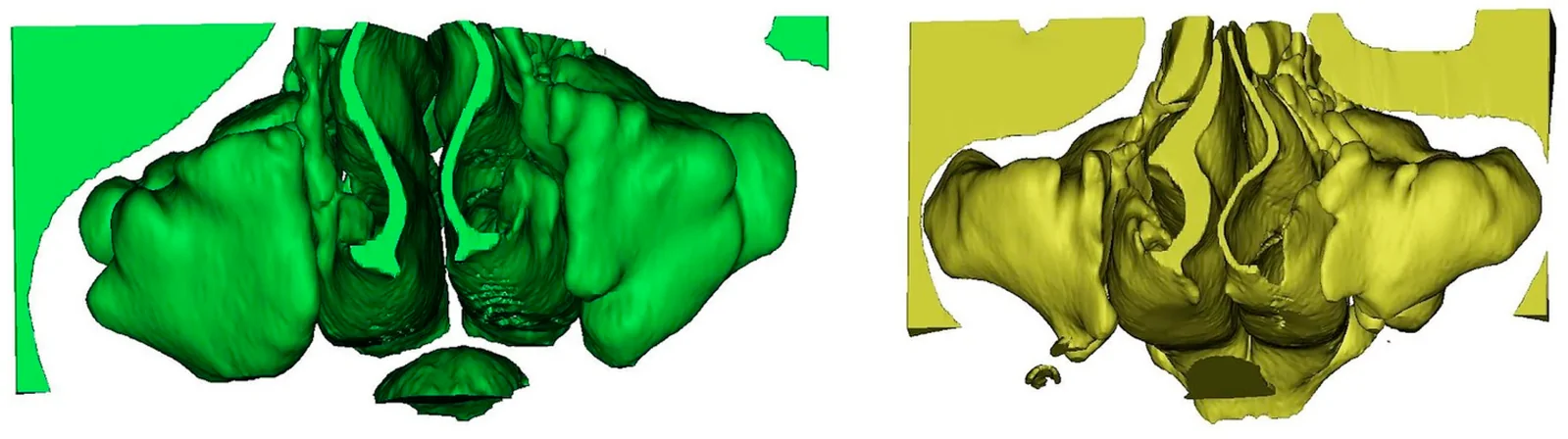
Representative three-dimensional reconstructions of the aerated maxillary sinuses demonstrating variation in external lobulated morphology. Pole measurements were obtained by counting anatomically distinct outward convex projections along the external surface of the reconstructed aerated maxillary sinus. The number and distribution of poles varied among individuals and between the right and left sides. The green and yellow colors represent two different representative individuals, selected to illustrate the range of inter-individual variability in sinus morphology. In the anterior view, the left and right sides of the image correspond to the patient’s right and left maxillary sinuses, respectively.

Pole measurements were performed using three-dimensional reconstructions of the aerated maxillary sinus generated from the segmented air cavity. After three-dimensional reconstruction, the external surface of each maxillary sinus air cavity was systematically evaluated. A pole was defined as an anatomically distinct outward convex projection of the external surface of the reconstructed aerated maxillary sinus. The total number of poles was recorded separately for the right and left maxillary sinuses. Pole counting was performed to provide a quantitative description of the lobulated external morphology of the aerated maxillary sinus and to assess bilateral morphological variation and symmetry between the right and left sides (Figure 7).

### 2.5. Data Analysis

Data were analyzed using SPSS Statistics version 18.0 (IBM Corp., Armonk, NY, USA). Descriptive statistics were used to summarize demographic characteristics, three-dimensional maxillary sinus morphology patterns, ostium status, septa presence, septa elongation/location, dental root extension, and morphometric measurements. Categorical variables were reported as frequencies and percentages, and continuous variables were reported as means and standard deviations. Statistical significance was set at *p* < 0.05. Given the exploratory nature of the analyses, no formal adjustment for multiple comparisons was applied; *p* values were interpreted together with effect sizes and the consistency of findings across related analyses. Normality was assessed using the Shapiro–Wilk test. For paired right–left comparisons, paired-samples *t*-tests were used when side-to-side difference scores were normally distributed, and Wilcoxon signed-rank tests were used when normality was not met. McNemar tests were used for paired binary right–left categorical comparisons. Sex-based differences were examined using independent-samples *t*-tests or Mann–Whitney U tests for continuous variables and chi-square or Fisher’s exact tests for categorical variables, as appropriate. Associations between age and morphometric or septa length variables were examined using Spearman’s rho correlations. Because the original 18-category three-dimensional morphology classification was anatomically meaningful and several categories had small frequencies, morphology-related findings were summarized descriptively rather than tested inferentially. Cross-tabulations with counts and row percentages were used to describe the distribution of coronal septa presence, axial septa presence, and dental root extension across morphology patterns. Septa elongation/location findings were also summarized descriptively using valid observations for each septa variable, with the most frequent observed categories presented for readability. To evaluate right–left morphological asymmetry, absolute side-to-side difference scores were calculated for ostium-to-sinus-floor distance, axial longitudinal sinus length, axial transverse sinus length, and pole measurements, and compared between symmetric and asymmetric cases using Mann–Whitney U tests. Categorical right–left mismatch variables were also created for ostium status, coronal septa presence, dental root extension, and axial septa presence, and were compared by morphological symmetry status using chi-square or Fisher’s exact tests. Measurement reliability was assessed using intraclass correlation coefficients (ICCs). Intra-rater reliability compared the primary investigator’s first measurement with the repeated measurement obtained one month later, whereas inter-rater reliability compared the primary investigator’s first measurement with the second observer’s measurement. ICCs were calculated using a two-way mixed-effects model with absolute agreement and 95% confidence intervals; single-measure ICCs were used as the primary reliability estimates. Inter-rater reliability of the proposed 18-category three-dimensional maxillary sinus morphological classification was evaluated using unweighted Cohen’s kappa because the morphology categories were nominal and did not represent an ordinal scale. Right- and left-sided classifications were evaluated separately. The percentage of exact agreement between the two observers was also calculated. Given the retrospective design and use of available eligible CT scans, no post hoc power analysis was conducted. Results were interpreted using descriptive statistics, effect estimates, confidence intervals where applicable, and *p* values.

## 3. Results

**Figure 8:**
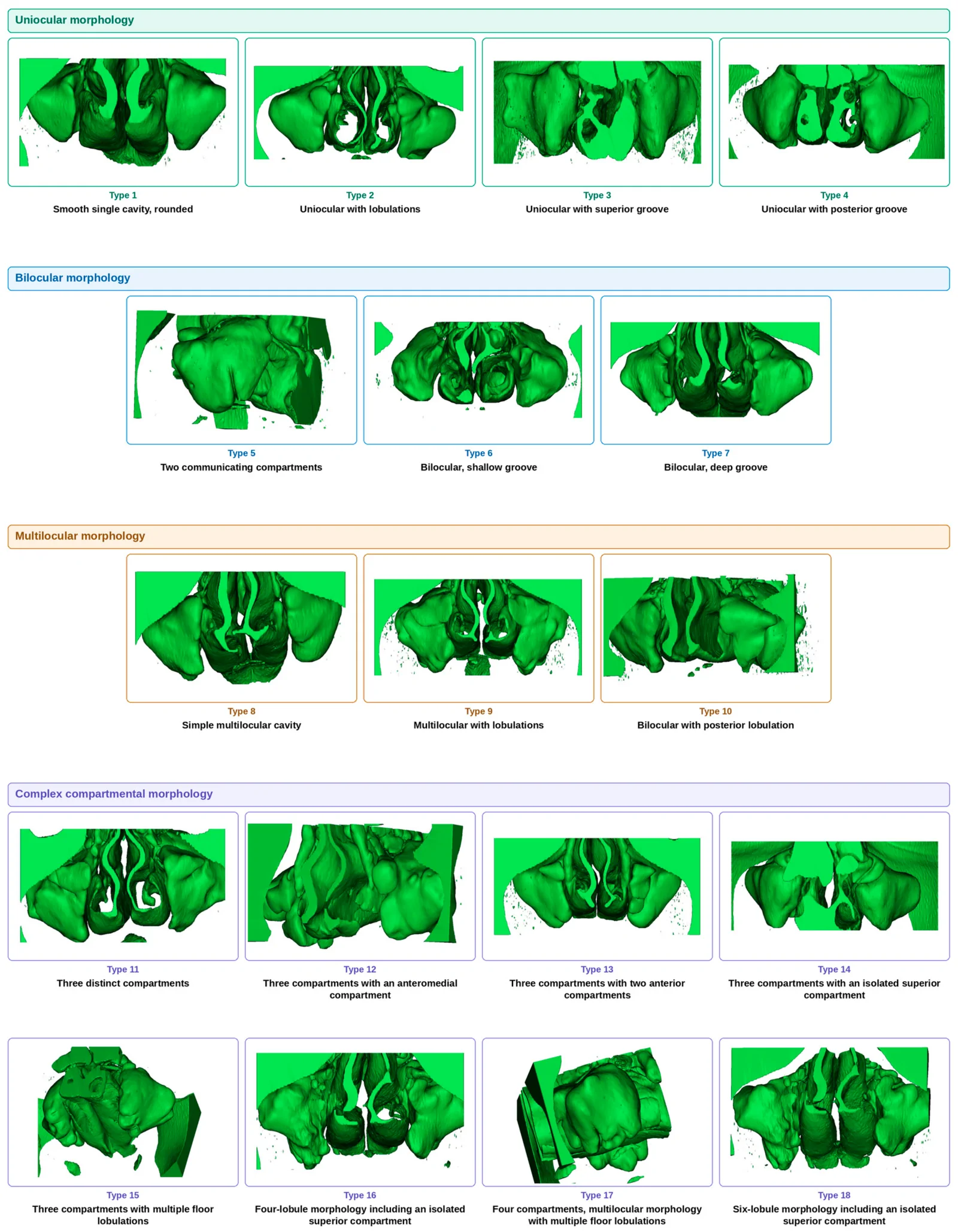
Three-dimensional reconstructions illustrating the proposed 18-subtype classification of the aerated maxillary sinus. Representative models are shown for each morphological subtype. Standard anterior (A) and posterior (P) views, automatically generated in Mimics, were used whenever these adequately demonstrated the defining anatomical characteristics. When necessary, lateral, inferior, or oblique views were selected to optimize visualization of the distinguishing morphological features. The viewing orientation for each panel is as follows: Types 1, 2, 6, 7, 8, 9, 11, 13, 16, and 18 are shown in the anterior view. Types 3, 4, and 14 in the posterior view. Type 5 and Type 17 in the posterolateral view. Type 10 in the anterolateral view. Type 12 in the anteromedial view. Type 15 in the posteroinferior view.

This retrospective morphometric study was designed to address several research questions. First, it aimed to determine whether three-dimensional maxillary sinus morphology and selected morphometric parameters differed between the right and left sides. Second, it investigated whether maxillary sinus morphometric parameters were associated with demographic characteristics, particularly sex and age. Third, the distribution of coronal septa, axial septa, and dental root extension across the original three-dimensional morphological patterns of the maxillary sinus was evaluated. Finally, the study examined whether right–left morphological asymmetry was associated with side-specific differences in sinus dimensions, ostium-to-sinus-floor distance, septal characteristics, and dental root extension. To facilitate visual interpretation of the proposed three-dimensional morphological classification, representative three-dimensional reconstructions were selected for each of the 18 morphological subtypes, illustrating the defining anatomical characteristics of each subtype and complementing the bilateral frequency distribution presented in Table 1 (Figure 8).

Before conducting the analyses addressing the study research questions, the distributional characteristics of the continuous variables were examined to guide the selection of appropriate statistical tests. Normality of continuous variables was assessed using the Shapiro–Wilk test. The main bilateral morphometric measurements, including ostium-to-sinus-floor distance and axial longitudinal and transverse sinus lengths, generally satisfied the normality assumption (Shapiro–Wilk *p* > 0.05). In contrast, age, right and left pole measurements, and most septa length variables deviated from normality. Therefore, parametric tests were considered appropriate for normally distributed morphometric measurements, whereas non-parametric tests were planned for non-normally distributed variables and septa-related measurements with limited available observations (Table 2). For paired right–left comparisons, normality of the side-to-side difference scores was assessed before selecting paired-samples *t*-tests or Wilcoxon signed-rank tests.

Research Question 1: Do three-dimensional maxillary sinus morphology and selected morphometric parameters differ between the right and left sides? Right-left comparisons were conducted to evaluate side-to-side differences in CT-based maxillary sinus morphometric parameters. The right ostium-to-sinus-floor distance was slightly greater than the left side (31.68 ± 5.09 mm vs. 31.01 ± 5.30 mm). This difference was statistically significant (t(124) = 1.981, *p* = 0.050); however, the corresponding effect size was small (Cohen’s d = 0.177), indicating that the magnitude of the side-to-side difference was limited. Wilcoxon signed-rank tests showed a significant right–left difference in axial longitudinal sinus length, Z = −2.214, *p* = 0.027, with the left side showing slightly higher values than the right side. No significant right–left difference was observed for axial transverse sinus length, Z = −0.341, *p* = 0.733. A significant side-to-side difference was also observed for pole measurements, Z = −3.638, *p* < 0.001, with right pole measurements higher than left pole measurements (Table 3).

Categorical right–left comparisons were then examined for ostium status, coronal septa presence, tooth extension, and axial septa presence. The right maxillary ostium was present in all cases. On the left side, the ostium was present in 113 cases (90.4%). The natural maxillary ostium was not visualized in two left maxillary sinuses (1.6%), whereas it appeared covered in 10 cases (8.0%). Because the right ostium showed no variability, ostium status was summarized descriptively rather than tested inferentially. McNemar tests showed no statistically significant right–left difference in coronal septa presence (right present: 46/124, 37.1%; left present: 42/124, 33.9%; *p* = 0.608). Similarly, axial septa presence did not differ significantly between the right and left sides (right present: 46/125, 36.8%; left present: 45/125, 36.0%; *p* = 1.000). In contrast, tooth extension into the sinus cavity differed significantly by side. Tooth extension was observed more frequently on the left side (20/125, 16.0%) than on the right side (8/125, 6.4%), with McNemar’s test indicating a statistically significant side-to-side difference (*p* = 0.002) (Table 4).

Research Question 2: Are maxillary sinus morphometric parameters associated with demographic characteristics, particularly sex and age? Sex-based differences in maxillary sinus morphometric parameters were evaluated using independent-sample *t*-tests for normally distributed variables and Mann–Whitney U tests for non-normally distributed variables. For the *t*-tests, Levene’s test was used to determine whether equal variances could be assumed. Male participants had significantly greater ostium-to-sinus-floor distances on both sides. Specifically, the right ostium-to-sinus-floor distance was significantly higher in males than in females (34.50 ± 4.08 mm vs. 28.99 ± 4.48 mm), t(123) = 7.177, *p* < 0.001, and the left ostium-to-sinus-floor distance was also significantly higher in males (33.82 ± 4.99 mm vs. 28.34 ± 4.09 mm), t(116.149) = 6.704, *p* < 0.001. Regarding axial sinus dimensions, males had significantly greater right axial transverse length, left axial longitudinal length, and left axial transverse length, whereas only modest differences were observed for the left axial longitudinal and transverse lengths. Right axial transverse length was significantly higher in males than in females (29.74 ± 4.76 mm vs. 25.99 ± 3.94 mm), t(123) = 4.815, *p* < 0.001. Left axial longitudinal length was slightly higher in males (38.13 ± 4.29 mm vs. 36.78 ± 2.90 mm), t(104.686) = 2.057, *p* = 0.042, as was left axial transverse length (28.70 ± 4.91 mm vs. 26.60 ± 3.80 mm), t(123) = 2.678, *p* = 0.008. In contrast, right axial longitudinal length did not differ significantly by sex, t(123) = 1.558, *p* = 0.122. For non-normally distributed variables, Mann–Whitney U tests showed no statistically significant sex-based differences in right or left pole measurements, right or left coronal septa 1 length, right or left axial septa length, or right or left sagittal septa length (all *p* > 0.05) (Table 5).

Age-related associations were examined using Spearman’s rho correlation coefficients. Among the main morphometric parameters, age showed a weak negative correlation with left axial transverse sinus length, indicating that older age was associated with slightly smaller left axial transverse measurements (ρ = −0.191, *p* = 0.032). No statistically significant correlations were observed between age and right or left pole measurements, right or left ostium-to-sinus-floor distance, right axial longitudinal or transverse sinus length, or left axial longitudinal sinus length. For septa length variables, age was not significantly correlated with right or left coronal septa 1 length, right or left axial septa length, or right or left sagittal septa length (all *p* > 0.05). Overall, age showed limited association with the evaluated CT-based maxillary sinus morphometric parameters, with only a weak negative correlation observed for left axial transverse sinus length.

Sex-based differences in categorical maxillary sinus findings were examined using chi-square tests or Fisher’s exact test when expected cell counts were small. Right–left morphological symmetry/asymmetry did not differ significantly by sex (*p* = 0.729). Left ostium status was similar between males and females and was interpreted descriptively because of sparse counts in the absent and covered categories. A statistically significant but modest sex-based difference was observed for right coronal septa presence. Right coronal septa were present in 28 of 61 males (45.9%) and 18 of 64 females (28.1%), χ^2^(1) = 4.244, *p* = 0.039, indicating that right coronal septa were more frequently observed in males. No significant sex-based difference was found for left coronal septa presence (*p* = 0.375), right or left tooth extension into the sinus cavity (*p* = 0.485 and *p* = 0.907, respectively), or right or left axial septa presence (*p* = 0.188 and *p* = 0.698, respectively).

Research Question 3: How are septa presence, axial septa presence, and dental root extension distributed across the original three-dimensional maxillary sinus morphology patterns? The most frequent right-sided morphology was 1-part unilocular/rounded morphology (69/125, 55.2%), followed by 1-part unilocular with lobulations (18/125, 14.4%) and 1-part multilocular with lobulations (15/125, 12.0%). A similar pattern was observed on the left side, where 1-part unilocular/rounded morphology was the most common pattern (67/125, 53.6%), followed by 1-part unilocular with lobulations (29/125, 23.2%) and 1-part multilocular with lobulations (9/125, 7.2%). Morphology-related findings were also summarized descriptively. On the right side, coronal septa were observed in 46 of 125 cases (36.8%), axial septa in 46 of 125 cases (36.8%), and tooth extension into the sinus cavity in eight of 125 cases (6.4%). Among the more frequently observed right-sided morphology patterns, septa-related findings were less common in the 1-part unilocular/rounded group than in more complex morphology groups. For example, right coronal septa were present in 17 of 69 cases (24.6%) with 1-part unilocular/rounded morphology, compared with 10 of 15 cases (66.7%) with 1-part multilocular with lobulations. Right axial septa showed a similar descriptive pattern, being present in 15 of 69 cases (21.7%) with 1-part unilocular/rounded morphology and 11 of 15 cases (73.3%) with 1-part multilocular with lobulations. Right tooth extension was uncommon overall but was most frequently observed in the 1-part multilocular with lobulations group (5/15, 33.3%). On the left side, coronal septa were observed in 42 of 124 cases (33.9%), axial septa in 45 of 125 cases (36.0%), and tooth extension into the sinus cavity in 20 of 125 cases (16.0%). Similar to the right side, septa-related findings were less frequent in the 1-part unilocular/rounded group and more frequent in several lobulated or complex morphology patterns. Left coronal septa were present in 13 of 66 cases (19.7%) with 1-part unilocular/rounded morphology and 16 of 29 cases (55.2%) with 1-part unilocular with lobulations. Left axial septa were present in 14 of 67 cases (20.9%) with 1-part unilocular/rounded morphology and 16 of 29 cases (55.2%) with 1-part unilocular with lobulations. Left tooth extension was observed in eight of 67 cases (11.9%) with 1-part unilocular/rounded morphology, six of 29 cases (20.7%) with 1-part unilocular with lobulations, and four of nine cases (44.4%) with 1-part multilocular with lobulations.

Septa elongation/location was coded using predefined anatomical location categories. Because several categories were infrequent or not observed for specific septa variables, septa elongation/location findings were summarized descriptively. For readability, the results table presents the most frequent observed locations for each septa variable, with less frequent observed locations grouped as “Other categories. For coronal septa, the most frequent location was the orbital wall. Among right coronal septa 1 cases, the orbital wall was the most common location (16/46, 34.8%), followed by orbital wall–lateral wall (11/46, 23.9%) and orbital wall–maxillary floor (6/46, 13.0%). Among left coronal septa 1 cases, the orbital wall was also the most common location (18/42, 42.9%), followed by orbital wall–lateral wall (7/42, 16.7%) and orbital wall–medial wall (6/42, 14.3%). Second coronal septa were uncommon, with right coronal septa 2 observed in six cases and left coronal septa 2 observed in three cases. For axial septa, the anterior wall was the most frequent location on both sides. Right axial septa were most commonly located at the anterior wall (22/44, 50.0%), followed by the lateral wall (8/44, 18.2%) and medial wall–lateral wall (6/44, 13.6%). Left axial septa were also most commonly located at the anterior wall (21/35, 60.0%), followed by medial wall–lateral wall (6/35, 17.1%) and anterior wall–medial wall (3/35, 8.6%). For sagittal septa, right-sided sagittal septa were most commonly located at the orbital wall (20/65, 30.8%), followed by the maxillary floor (15/65, 23.1%) and anterior wall–orbital wall (8/65, 12.3%). Left-sided sagittal septa were most commonly located at the anterior wall–orbital wall (18/65, 27.7%), followed by the orbital wall (15/65, 23.1%), maxillary floor (9/65, 13.8%), and tooth root (9/65, 13.8%).

Research Question 4: Is right–left morphological asymmetry associated with side-to-side differences in sinus dimensions, ostium-to-sinus-floor distance, septa-related features, and dental root extension? Absolute right–left difference scores were calculated to evaluate whether morphologically symmetric and asymmetric cases differed in the magnitude of side-to-side morphometric differences. Mann–Whitney U tests showed no statistically significant difference between symmetric and asymmetric cases in absolute right–left ostium-to-sinus-floor distance difference, axial longitudinal sinus length difference, or axial transverse sinus length difference. However, absolute right–left pole difference was greater in the asymmetric group than in the symmetric group, U = 643.500, Z = −3.515, *p* < 0.001. This finding suggests that cases classified as morphologically asymmetric also showed greater side-to-side difference in pole measurements.

To further evaluate right–left morphological asymmetry, categorical mismatch variables were created to identify whether right and left sides differed in ostium status, coronal septa presence, tooth extension, and axial septa presence. Coronal septa mismatch differed significantly by morphological symmetry status. Coronal septa mismatch was observed in two of 22 symmetric cases (9.1%) and 32 of 102 asymmetric cases (31.4%), χ^2^(1) = 4.515, *p* = 0.034, suggesting that morphologically asymmetric cases tended to exhibit side-to-side discordance in coronal septa presence more frequently than symmetric cases. No statistically significant differences were observed between symmetric and asymmetric cases for ostium status mismatch, tooth extension mismatch, or axial septa mismatch. Ostium mismatch was observed in one of 22 symmetric cases (4.5%) and 11 of 103 asymmetric cases (10.7%; Fisher’s exact *p* = 0.691). Tooth extension mismatch was observed in two of 22 symmetric cases (9.1%) and 12 of 103 asymmetric cases (11.7%; Fisher’s exact *p* = 1.000). Axial septa mismatch was observed in four of 22 symmetric cases (18.2%) and 31 of 103 asymmetric cases (30.1%), but this difference was not statistically significant, χ^2^(1) = 1.277, *p* = 0.259.

### Intra-Rater and Inter-Rater Reliability of CT-Based Morphometric Measurements

Measurement reliability was evaluated using single-measure intraclass correlation coefficients (ICCs) based on a two-way mixed-effects model with absolute agreement. Intra-rater reliability, assessed by comparing the primary investigator’s first measurement with the repeated measurement obtained one month later, showed high to excellent agreement across all morphometric measurements. Intra-rater ICCs ranged from 0.891 for right ostium-to-sinus-floor distance to 0.972 for left ostium-to-sinus-floor distance. Inter-rater reliability, assessed by comparing the primary investigator’s first measurement with the second observer’s measurement, was excellent across all measurements, with ICCs ranging from 0.984 to 0.992. All ICCs were statistically significant at *p* < 0.001 (Table 6). Inter-rater reliability of the proposed 18-category three-dimensional morphological classification was substantial. For the right maxillary sinus, the two observers agreed on 101 of 125 classifications (80.8%), yielding a Cohen’s κ of 0.726 (*p* < 0.001). For the left maxillary sinus, the observers also agreed on 101 of 125 classifications (80.8%), yielding a Cohen’s κ of 0.717 (*p* < 0.001).

## 4. Discussion

The present study characterized the three-dimensional morphology of the aerated maxillary sinus using CT-based air-cavity reconstruction and identified a spectrum of morphological configurations within the study population. Unilocular cavities represented the most common morphological pattern, whereas bilocular and multilocular configurations were observed less frequently. Rather than representing entirely separate anatomical entities, these patterns may reflect varying degrees of internal morphological complexity within the aerated maxillary sinus. The predominance of relatively simple unilocular configurations, together with the presence of increasingly complex lobulated and compartmentalized cavities, suggests that maxillary sinus morphology may be better interpreted as a continuum of three-dimensional anatomical variation than as a limited number of discrete morphological categories. This interpretation should be considered descriptive and requires further investigation in different populations and imaging datasets. Previous studies using three-dimensional CT- or CBCT-based segmentation have mainly concentrated on volumetric evaluation, segmentation methodology or algorithm validation, rather than the detailed characterization of the internal structure of the aerated maxillary sinus [2,13]. While these studies have demonstrated the feasibility and clinical utility of three-dimensional reconstruction, they have not provided a standardized descriptive framework for the internal architecture of the aerated sinus cavity [2,13]. In contrast, the present study uses CT-based air-cavity reconstruction as a tool for anatomical investigation and proposes a reproducible three-dimensional morphological classification based on internal compartmentalization, lobulations, grooves, and isolated compartments. Unlike many previous CBCT-based investigations, the present methodology employed medical CT datasets and a predefined Hounsfield Unit threshold in Mimics software for standardized air-cavity segmentation, thereby providing a reproducible basis for three-dimensional anatomical evaluation. This approach may complement conventional morphometric evaluation by providing additional anatomical insight into the three-dimensional organization of the aerated maxillary sinus.

Conventional anatomical descriptions have generally portrayed the maxillary sinus as a pyramidal or triangular cavity, while many radiological studies have focused primarily on linear measurements, sinus volume, or isolated anatomical variations [7,21]. In the present study, three-dimensional air-cavity reconstruction provided additional visualization of the internal configuration of the aerated cavity, including lobulations, grooves, and compartment-like extensions that may not be readily appreciated on conventional two-dimensional assessment.

Within this context, features such as prominent superior or posterior grooves, isolated superior compartments, and multiple surface lobulations may be interpreted as components contributing to this spectrum of morphological complexity rather than as entirely separate anatomical entities.

By characterizing the internal configuration of the aerated cavity, this approach may complement conventional morphometric and septal evaluations and provide additional information regarding individual anatomical variability. Consistent with the findings of the present study, unilocular configurations were the most frequently observed pattern, whereas increasing internal complexity was associated with a range of lobulated and compartmentalized morphologies. These observations suggest that three-dimensional air-cavity reconstruction may complement conventional anatomical descriptions by providing additional information regarding the internal configuration of the aerated maxillary sinus.

An additional finding of the present study was the observed relationship between three-dimensional sinus morphology and the distribution of internal septa. Coronal septa were identified more frequently in morphologically complex or lobulated sinus configurations than in relatively simple unilocular cavities. For example, coronal septa were present in approximately 24.6% of rounded unilocular sinuses, whereas this proportion increased to 66.7% in multilocular lobulated configurations. Although this study was not designed to investigate developmental mechanisms, these observations suggest that septa may contribute to the internal morphological configuration of the aerated cavity by being associated with grooves, partial partitions, and compartment-like extensions [10].

Previous studies have primarily focused on the prevalence, orientation, and developmental origin of maxillary sinus septa, particularly Underwood septa, because of their importance in sinus floor augmentation and the prevention of Schneiderian membrane perforation [5,9,18,24]. While these investigations have provided valuable information for surgical planning, they have generally evaluated septa as individual anatomical structures [5,6]. In contrast, three-dimensional air-cavity reconstruction allows septa to be considered within the overall internal morphology of the aerated sinus cavity, providing additional anatomical context for their spatial distribution and relationship with adjacent morphological features [10]. Notably, although the functional implications of these observations were not investigated, this morphological approach may contribute to a more comprehensive anatomical description of individual maxillary sinus variability and may provide useful information for future radiological, anatomical, and preoperative studies.

A further observation of the present study was that axial septa showed a distribution pattern similar to that of coronal septa, with a higher frequency in morphologically more complex sinus configurations. Although axial septa have received less attention than Underwood or coronal septa in the existing literature, they may represent an additional source of internal anatomical variation [5,9,10,24]. In the present study, axial septa were most frequently identified along the anterior wall on both sides and were more commonly observed in multilobulated configurations. These findings may suggest that internal morphological variation in the maxillary sinus is not confined to a single anatomical plane but may involve multiple spatial orientations. However, the developmental mechanisms underlying these septal configurations were beyond the scope of the present study and warrant further anatomical investigation.

Dental root extension into the maxillary sinus also demonstrated variation among different morphological patterns. The close anatomical relationship between the posterior maxillary dentition and the sinus floor has been widely recognized because of its relevance to implant dentistry, endodontic surgery, and odontogenic maxillary sinus disease [9,25,26,27]. In the present study, dental root extension was observed more frequently on the left side and tended to occur more often in morphologically complex sinus configurations. Although no causal relationship can be inferred from these findings, the combined evaluation of three-dimensional sinus morphology and dental root extension may provide additional anatomical information regarding individual maxillary sinus variability. This integrated anatomical assessment may be useful for future radiological and anatomical investigations and could potentially complement preoperative evaluation in selected clinical settings.

Another finding of the present study was the generally similar distribution of three-dimensional morphological patterns between the right and left maxillary sinuses. Statistically significant side-to-side differences were identified for selected morphometric parameters, including the ostium-to-sinus-floor distance (*p* = 0.050), axial longitudinal sinus length (*p* = 0.027) and pole measurements (*p* < 0.001). However, the difference observed for the ostium-to-sinus-floor distance was associated with a small effect size and should therefore be interpreted cautiously. In addition, dental root extension was observed more frequently on the left side (16.0%) than on the right side (6.4%). These findings are consistent with previous reports indicating that, although the paranasal sinuses often exhibit an overall bilateral similarity, individual anatomical variation between sides is relatively common [1]. Accordingly, the present findings may support the value of side-specific three-dimensional anatomical assessment rather than assuming complete bilateral equivalence during radiological evaluation.

The present study also highlights the potential value of CT-based three-dimensional air-cavity reconstruction for anatomical evaluation of the maxillary sinus. Conventional multiplanar CT remains the standard imaging modality for assessing sinonasal anatomy; however, image interpretation is primarily based on sequential two-dimensional sections [27,28]. Three-dimensional reconstruction may complement conventional assessment by providing a more comprehensive visualization of the aerated cavity, including lobulations, grooves, compartment-like extensions, and superior compartments that may be less readily appreciated on routine multiplanar evaluation [1,2,6].

It is also important to distinguish the objective of the present study from recent developments in automated image segmentation. Previous investigations, including those by Morgan et al., have demonstrated considerable progress in artificial-intelligence-based maxillary sinus segmentation using convolutional neural networks [2,3]. These studies have primarily focused on improving segmentation accuracy, automation, and computational performance [2]. In contrast, the present study used CT-based segmentation as an anatomical reconstruction tool rather than as the primary subject of investigation [2]. Within this framework, segmentation enabled systematic evaluation of the three-dimensional morphology of the aerated maxillary sinus together with its relationship to selected morphometric parameters and anatomical features.

The selection of the upper Hounsfield Unit threshold for air-cavity segmentation varies across previous studies [2,22,23]. Different upper threshold values have been reported depending on the imaging modality, anatomical region, segmentation protocol, and software, generally ranging from approximately −300 HU to −200 HU [2,22,23]. For example, Morgan et al. used a threshold range of −1024 to −200 HU for semi-automatic segmentation of the maxillary sinus, whereas Lee et al. employed −1024 to −260 HU for three-dimensional reconstruction of the sphenoid sinus using Mimics software [2,23]. Similarly, Cherobin et al. suggested that the optimal upper threshold may depend on CT scanner characteristics, acquisition protocols, anatomical region, and patient-specific anatomy, indicating that no single threshold is universally applicable to all segmentation tasks [22]. Based on these previous studies, the threshold range of −1024 to −259 HU was selected for the present study and applied consistently to all CT datasets without individual adjustment, thereby ensuring a standardized segmentation protocol across the study population.

The natural maxillary ostium was identified in all right maxillary sinuses, whereas a small number of left-sided ostia were classified as either not visualized or covered. As congenital absence of the natural maxillary ostium is extremely rare, cases recorded as not visualized in the present study most likely represent radiological non-visualization rather than true anatomical absence. Similarly, ostia classified as covered were considered to be obscured by adjacent soft tissue or mucosal coverage on CT imaging. These findings may reflect normal anatomical variation or limitations of CT visualization and should therefore be interpreted with caution.

The present study was designed as a descriptive anatomical investigation rather than a functional analysis. Nevertheless, the observed variation in the three-dimensional morphology of the aerated maxillary sinus may be relevant to future studies investigating sinonasal physiology [11,29]. Previous computational fluid dynamics studies have suggested that airflow patterns and ventilation within the paranasal sinuses are influenced by cavity geometry and ostial configuration [11,29,30]. Within this context, the multilobulated and compartmentalized configurations identified in the present study may represent anatomical variations in interest.

The three-dimensional morphological classification proposed in this study should be considered a descriptive anatomical framework rather than a predictive clinical tool. Future studies may evaluate its reproducibility across different populations and imaging modalities, as well as investigating potential associations with surgical outcomes, sinus augmentation procedures, implant planning and endoscopic interventions. Additionally, incorporating this classification into computational fluid dynamics models could clarify whether specific patterns of internal compartmentalization and aerated cavity morphology influence sinus ventilation or airflow characteristics. Such investigations may also help to determine the potential clinical utility of the proposed classification. Likewise, describing the maxillary sinus according to the morphology of its aerated cavity, in addition to conventional morphometric measurements, may contribute to a more comprehensive anatomical characterization of individual sinus variability [11,19,29,30,31]. Further multidisciplinary studies combining three-dimensional morphological assessment with functional imaging, computational modeling and clinical outcome data may further clarify the physiological and clinical relevance of these anatomical patterns.

Several limitations should be considered when interpreting the findings of the present study. First, this was a retrospective, single-center investigation, which may limit the generalizability of the results to other populations. Second, although the proposed morphological classification enabled a detailed description of three-dimensional air-cavity configurations, several less common morphological patterns were represented by relatively few cases, limiting statistical comparisons for these subgroups. Moreover, the exploratory analyses involved multiple comparisons without formal adjustment; therefore, findings with marginal *p* values should be interpreted cautiously and validated in independent samples. Third, the selected threshold range (−1024 to −259 HU) may have influenced the reconstructed morphology because the precise air-soft tissue boundary on CT is not uniformly defined. Depending on the imaging characteristics and local anatomy, partial inclusion of thin mucosal tissues or exclusion of small air spaces cannot be completely excluded. Although the threshold range was selected based on previously published segmentation protocols and applied consistently to all datasets, we did not perform a sensitivity analysis using alternative threshold values. Future studies should evaluate the influence of different segmentation thresholds on morphometric parameters and three-dimensional surface morphology to further establish the robustness of CT-based maxillary sinus reconstruction.

Fourth, the present study was designed as a descriptive anatomical investigation; therefore, functional parameters such as airflow dynamics, mucociliary clearance, computational fluid dynamics, and surgical outcomes were not evaluated. In addition, although CT provides excellent visualization of osseous anatomy, radiological assessment alone cannot fully account for all mucosal or inflammatory conditions that may influence the appearance of the aerated cavity. Furthermore, all examinations were acquired using a single CT scanner and a standardized imaging protocol, which ensured methodological consistency but may limit the generalizability of the findings to other CT systems. Although three-dimensional segmentation enables volumetric assessment, volumetric analysis was intentionally not performed because the primary objective of the present study was to characterize the three-dimensional morphology of the aerated maxillary sinus rather than sinus volume. Future multicenter studies incorporating larger cohorts, automated segmentation techniques, and functional analyses may help to further investigate the anatomical and potential physiological relevance of the morphological patterns identified in the present study.

From a clinical perspective, this classification may complement conventional morphometric assessment by providing a more comprehensive description of the three-dimensional anatomy of the aerated maxillary sinus. Such characterization may facilitate standardized reporting of anatomical variation and provide a reproducible anatomical framework for future radiological investigations, computational fluid dynamics studies, artificial intelligence applications, and preoperative surgical planning.

## 5. Conclusions

This study described the three-dimensional morphology of the aerated maxillary sinus using CT-based air-cavity reconstruction combined with standardized morphometric analysis. The aerated cavity demonstrated a spectrum of morphological configurations, ranging from relatively simple unilocular patterns to more complex lobulated and compartmentalized structures. In addition to conventional morphometric measurements, three-dimensional reconstruction enabled systematic evaluation of septal characteristics, bilateral symmetry, and dental root extension within the same anatomical framework.

The findings suggest that characterization of the aerated cavity may complement conventional anatomical and morphometric assessment by providing additional information regarding individual variability in maxillary sinus morphology. Rather than relying solely on linear dimensions or sinus volume, three-dimensional air-cavity reconstruction offers an alternative perspective for describing the internal organization of the aerated space. Although this study was designed as a descriptive anatomical investigation, the proposed morphological characterization may provide a useful reference for future radiological, anatomical, and computational studies. Further investigations integrating three-dimensional morphology with functional imaging, computational fluid dynamics, and clinical outcomes may help clarify the potential physiological and clinical relevance of these anatomical variations.

## Figures and Tables

**Table 1:** Classification of three-dimensional morphological patterns of the aerated maxillary sinus based on the anatomical variations observed in the present study.

Main Category	Subtype	Morphological Characteristics	Right Sinus *n* (%)	Left Sinus *n* (%)
Uniocular morphology	Type 1	Smooth single cavity with rounded configuration; may show unilateral size predominance or dental root protrusion	69 (55.2)	67 (53.6)
	Type 2	Uniocular cavity with anterior, posterior, superior, or multiple lobulations	18 (14.4)	29 (23.2)
	Type 3	Uniocular cavity with shallow or deep superior groove, with a small lobulation	1 (0.8)	2 (1.6)
	Type 4	Uniocular cavity with shallow groove	6 (4.8)	1 (0.8)
Bilocular morphology	Type 5	Two communicating compartments	3 (2.4)	4 (3.2)
	Type 6	Bilocular cavity separated by a shallow groove	2 (1.6)	2 (1.6)
	Type 7	Bilocular cavity separated by a deep groove, occasionally with a small superior compartment	5 (4.0)	5 (4.0)
Multilocular morphology	Type 8	Simple multilocular cavity	0 (0.0)	1 (0.8)
	Type 9	Multilocular cavity with posterior, lateral, floor, or multiple lobulations	15 (12.0)	9 (7.2)
	Type 10	Bilocular morphology with a prominent posterior lobulation	1 (0.8)	0 (0.0)
Complex compartmental morphology	Type 11	Three distinct compartments	1 (0.8)	2 (1.6)
	Type 12	Three compartments with an additional anteromedial compartment	0 (0.0)	1 (0.8)
	Type 13	Three compartments with two anterior compartments	1 (0.8)	0 (0.0)
	Type 14	Three compartments including an isolated superior compartment	1 (0.8)	0 (0.0)
	Type 15	Three compartments with multiple floor lobulations	1 (0.8)	0 (0.0)
	Type 16	Four-lobule morphology including an isolated superior compartment	0 (0.0)	1 (0.8)
	Type 17	Four-compartment multilocular morphology with multiple floor lobulations	0 (0.0)	1 (0.8)
	Type 18	Six-lobule morphology including an isolated superior compartment	1 (0.8)	0 (0.0)

**Table 2:** Descriptive statistics and normality assessment of continuous variables.

Variable	n	Mean ± SD	Median	Min–Max	Shapiro–Wilk *p*
Age	125	37.04 ± 15.37	33.00	12–81	<0.001
Right maxillary sinus poles	125	6.94 ± 2.22	6.00	1–14	<0.001
Left maxillary sinus poles	125	6.34 ± 2.00	6.00	1–12	<0.001
Right ostium-to-sinus-floor distance, coronal plane (mm)	125	31.68 ± 5.09	31.85	17.82–40.87	0.083
Left ostium-to-sinus-floor distance, coronal plane (mm)	125	31.01 ± 5.30	30.68	14.80–42.85	0.098
Right axial longitudinal sinus length (mm)	125	36.89 ± 4.12	37.30	23.55–48.23	0.053
Right axial transverse sinus length (mm)	125	27.82 ± 4.73	27.81	15.18–39.71	0.795
Left axial longitudinal sinus length (mm)	125	37.44 ± 3.69	37.61	27.32–47.15	0.117
Left axial transverse sinus length (mm)	125	27.63 ± 4.49	27.80	14.85–38.80	0.988
Right coronal septa 1 length (mm)	37	11.14 ± 6.50	8.86	5.57–37.98	<0.001
Right coronal septa 2 length (mm)	5	8.46 ± 4.75	6.64	4.20–16.36	0.236
Left coronal septa 1 length (mm)	39	10.18 ± 4.06	9.11	4.30–23.24	0.004
Left coronal septa 2 length (mm)	4	5.47 ± 1.49	5.47	3.71–7.24	0.999
Right axial septa length (mm)	48	8.35 ± 3.21	7.41	3.52–15.59	0.014
Left axial septa length (mm)	43	9.17 ± 5.88	8.05	2.70–40.38	<0.001
Right sagittal septa length (mm)	65	9.81 ± 4.74	8.73	4.07–24.63	<0.001
Left sagittal septa length (mm)	69	11.39 ± 5.37	10.57	3.59–28.72	<0.001

Note. Normality was assessed using the Shapiro–Wilk test. Variables with *p* > 0.05 were considered approximately normally distributed. Although the Shapiro–Wilk test was non-significant for the second coronal septa length variables, these variables were interpreted descriptively because of very small sample sizes.

**Table 3:** Right–left comparisons of maxillary sinus morphometric parameters.

Parameter	Right Side	Left Side	Test Statistic	*p* Value	Interpretation
Ostium-to-sinus-floor distance, coronal plane (mm) *	31.68 ± 5.09	31.01 ± 5.30	t(124) = 1.981	0.050	Right > Left
Axial longitudinal sinus length (mm) **	36.89 ± 4.12; Median = 37.30	37.44 ± 3.69; Median = 37.61	Z = −2.214	0.027	Left > Right
Axial transverse sinus length (mm) **	27.82 ± 4.73; Median = 27.81	27.63 ± 4.49; Median = 27.80	Z = −0.341	0.733	No significant difference
Pole measurement **	6.94 ± 2.22; Median = 6.00	6.34 ± 2.00; Median = 6.00	Z = −3.638	<0.001	Right > Left

Note. Values are presented as mean ± standard deviation unless otherwise indicated. * The paired-samples *t*-test was used for ostium-to-sinus-floor distance because the side-to-side difference score met the normality assumption. ** Wilcoxon signed-rank tests were used for variables whose side-to-side difference scores did not meet the normality assumption.

**Table 4:** Right–left distribution and paired comparison of categorical maxillary sinus findings.

Variable	Category	Right Side, n/N (%)	Left Side, n/N (%)	Paired Distribution	*p* Value
Ostium status	Present	125/125 (100.0%)	113/125 (90.4%)	R present/L present = 113; R present/L absent = 2; R present/L covered = 10	—
	Absent	0/125 (0.0%)	2/125 (1.6%)		
	Covered	0/125 (0.0%)	10/125 (8.0%)		
Coronal septa presence	Present	46/124 (37.1%)	42/124 (33.9%)	Both present = 27; R only = 19; L only = 15; both absent = 63	0.608
	Absent	78/124 (62.9%)	82/124 (66.1%)		
Tooth extension into sinus cavity	Present	8/125 (6.4%)	20/125 (16.0%)	Both present = 7; R only = 1; L only = 13; both absent = 104	0.002
	Absent	117/125 (93.6%)	105/125 (84.0%)		
Axial septa presence	Present	46/125 (36.8%)	45/125 (36.0%)	Both present = 28; R only = 18; L only = 17; both absent = 62	1.000
	Absent	79/125 (63.2%)	80/125 (64.0%)		

Note. Values are presented as n/N (%). McNemar tests were used for paired binary right–left comparisons. Ostium status was summarized descriptively because the right ostium was present in all cases; therefore, inferential paired testing was not performed for ostium status. Coronal septa analyses were based on 124 paired observations because coronal septa could not be reliably assessed in one case. R = right; L = left.

**Table 5:** Sex-based differences in maxillary sinus morphometric parameters.

Parameter	Male	Female	Test Statistic	*p* Value	Effect/Difference
Right ostium-to-sinus-floor distance (mm) *	34.50 ± 4.08	28.99 ± 4.48	t(123) = 7.177	<0.001	Mean difference = 5.51; Cohen’s d = 1.284
Left ostium-to-sinus-floor distance (mm) *	33.82 ± 4.99	28.34 ± 4.09	t(116.149) = 6.704	<0.001	Mean difference = 5.48; Cohen’s d = 1.205
Right axial longitudinal sinus length (mm) *	37.47 ± 4.68	36.33 ± 3.44	t(123) = 1.558	0.122	Mean difference = 1.14; Cohen’s d = 0.279
Right axial transverse sinus length (mm) *	29.74 ± 4.76	25.99 ± 3.94	t(123) = 4.815	<0.001	Mean difference = 3.75; Cohen’s d = 0.862
Left axial longitudinal sinus length (mm) *	38.13 ± 4.29	36.78 ± 2.90	t(104.686) = 2.057	0.042	Mean difference = 1.35; Cohen’s d = 0.371
Left axial transverse sinus length (mm) *	28.70 ± 4.91	26.60 ± 3.80	t(123) = 2.678	0.008	Mean difference = 2.10; Cohen’s d = 0.479
Right maxillary sinus poles **	n = 61; mean rank = 65.42	n = 64; mean rank = 60.70	U = 1804.500; Z = −0.752	0.452	
Left maxillary sinus poles **	n = 61; mean rank = 63.66	n = 64; mean rank = 62.37	U = 1911.500; Z = −0.207	0.836	
Right coronal septa 1 length (mm) **	n = 21; mean rank = 17.10	n = 16; mean rank = 21.50	U = 128.000; Z = −1.226	0.220	
Left coronal septa 1 length (mm) **	n = 21; mean rank = 18.07	n = 18; mean rank = 22.25	U = 148.500; Z = −1.141	0.254	
Right axial septa length (mm) **	n = 26; mean rank = 24.42	n = 22; mean rank = 24.59	U = 284.000; Z = −0.041	0.967	
Left axial septa length (mm) **	n = 23; mean rank = 22.85	n = 20; mean rank = 21.03	U = 210.500; Z = −0.475	0.635	
Right sagittal septa length (mm) **	n = 33; mean rank = 33.61	n = 32; mean rank = 32.38	U = 508.000; Z = −0.262	0.793	
Left sagittal septa length (mm) **	n = 35; mean rank = 36.40	n = 34; mean rank = 33.56	U = 546.000; Z = −0.588	0.556	

Note. Values for independent-samples *t*-tests are presented as mean ± standard deviation. Values for Mann–Whitney U tests are presented as n and mean rank. * Independent-sample *t*-test. ** Mann–Whitney U test. When Levene’s test was significant, the equal variances not assumed result was reported.

**Table 6:** Intra-rater and inter-rater reliability of CT-based morphometric measurements.

Measurement	Intra-Rater ICC	95% CI	*p* Value	Inter-Rater ICC	95% CI	*p* Value
Right ostium-to-sinus-floor distance	0.891	0.805–0.934	<0.001	0.991	0.983–0.994	<0.001
Left ostium-to-sinus-floor distance	0.972	0.457–0.992	<0.001	0.992	0.985–0.996	<0.001
Right axial longitudinal sinus length	0.941	0.664–0.978	<0.001	0.988	0.967–0.994	<0.001
Right axial transverse sinus length	0.964	0.477–0.990	<0.001	0.992	0.988–0.995	<0.001
Left axial longitudinal sinus length	0.938	0.104–0.984	<0.001	0.984	0.927–0.993	<0.001
Left axial transverse sinus length	0.958	0.292–0.989	<0.001	0.987	0.972–0.993	<0.001

Note. ICC = intraclass correlation coefficient; CI = confidence interval. Single-measure ICCs are reported. Intra-rater reliability compared the primary investigator’s first measurement with the repeated measurement obtained one month later. Inter-rater reliability compared the primary investigator’s first measurement with the second observer’s measurement. ICCs were calculated using a two-way mixed-effects model with absolute agreement.

## Data Availability

We confirm that the main data supporting this study’s findings are available within the article, and any additional data are available upon request from the corresponding author. The anonymized morphometric dataset, segmentation-derived measurements, and additional methodological details supporting the findings of this study are available upon reasonable request. Because the study is based on retrospective clinical CT images obtained from a hospital archive, the original DICOM datasets and complete three-dimensional reconstructions cannot be made publicly available due to institutional ethics regulations and patient privacy considerations. Requests for additional anonymized data should be submitted by e-mail to the corresponding author, and access will be considered according to the purpose of the request and applicable institutional ethics and data-sharing regulations.

## Abbreviations

The following abbreviations are used in this manuscript:

MS	Maxillary sinus
CT	Computed Tomography
3D	Three-dimensional
HU	Hounsfield unit
OSFD	Ostium-to-sinus-floor distance
